# microRNA-99a Restricts Replication of Hepatitis C Virus by Targeting mTOR and De Novo Lipogenesis

**DOI:** 10.3390/v12070696

**Published:** 2020-06-27

**Authors:** Eun Byul Lee, Pil Soo Sung, Jung-Hee Kim, Dong Jun Park, Wonhee Hur, Seung Kew Yoon

**Affiliations:** 1 The Catholic University Liver Research Center, College of Medicine, The Catholic University of Korea, Seoul 06591, Korea; blue00w@catholic.ac.kr (E.B.L.); pssung@catholic.ac.kr (P.S.S.); kim.jh@catholic.ac.kr (J.-H.K.); akhpdyj@catholic.ac.kr (D.J.P.); wendyhur@catholic.ac.kr (W.H.); 2 Division of Gastroenterology and Hepatology, Department of Internal Medicine, College of Medicine, Seoul St. Mary’s Hospital, The Catholic University of Korea, Seoul 06591, Korea

**Keywords:** hepatitis C virus, microRNA-99a, lipid droplet, mTOR, viral replication

## Abstract

In this study, we investigated the role of microRNA-99a (miR-99a) in hepatitis C virus (HCV) replication and lipogenesis in hepatocytes. Cell-culture-derived HCV (HCVcc) infection caused down-regulation of miR-99a in Huh-7 cells, and the relative levels of miR-99a were significantly lower in the sera of the HCV-infected patients than in those of healthy controls. Transfection of miR-99a-5p mimics resulted in a decrease in the intracellular and secreted HCV RNA levels. It also caused a decreased mammalian target of rapamycin (mTOR) protein level and phosphorylation of its downstream targets in HCV-replicating cells. Sterol regulatory element binding protein (SREBP)-1c expression and intracellular lipid accumulation decreased when either miR-99a-5p mimics or si-mTOR was transfected in oleic acid-treated Huh-7 cells. Overexpression of mTOR rescued HCV RNA replication and lipid droplet accumulation in miR-99a-5p mimics-transfected HCV replicon cells. Our data demonstrated that miR-99a ameliorates intracellular lipid accumulation by regulating mTOR/SREBP-1c and causes inefficient replication and packaging of intracellular HCV.

## 1. Introduction

Worldwide, approximately 71 million people are infected with hepatitis C virus (HCV), and they have an increased risk of liver cirrhosis and hepatocellular carcinoma (HCC) [1,2,3]. The viral life cycle of HCV strongly depends on intracellular lipid droplets (LDs) [4]. Newly synthesized viral RNA genomes are recruited to endoplasmic reticular membranes that surround LDs, and LDs are involved in the viral packing step in HCV production [4]. Host lipid architectures and molecules involved in lipid metabolism are closely associated with the HCV life cycle [4,5].

The mammalian target of rapamycin (mTOR) signaling pathway is one of the major cellular pathways involved in the regulation of HCV infection [6]. mTOR is a key component of two structurally and functionally distinct protein complexes (mTORC1 and mTORC2) that regulate cell signaling pathways by phosphorylating several downstream targets [7]. mTOR directly phosphorylates the ribosomal protein S6 kinases (S6K1 and S6K2) and eukaryotic initiation factor 4E (eIF4E)-binding protein (4E-BP1 and 4E-BP2), which control specific steps in the initiation of cap-dependent translation [7]. Portsmann et al. identified the role of mTORC1 in lipogenesis and found that rapamycin blocked the expression of genes involved in lipogenesis, impairing nuclear accumulation of the sterol regulatory element binding proteins (SREBPs) [8]. However, there have been no studies that identified the factors used by HCV to regulate mTOR activity and lipid biogenesis. In this study, we studied the role of miRNA-99a (miR-99a) in HCV replication and lipogenesis via mTOR regulation in hepatocytes.

## 2. Materials and Methods

### 2.1. Serum Samples of the Patients

A total of 37 patients with chronic HCV infection (19 patients with genotype 1b, 18 patients with genotype 2a) and 14 healthy controls were recruited for this study. The patients did not receive direct-acting antivirals or pegylated interferon-based treatments. This study was approved by the institutional review board of Seoul St. Mary’s Hospital (KC15TISI0605, approval date: 28 July 2017). We followed the ethical standards of the responsible committee on human experimentation and the Declaration of Helsinki, 1975.

### 2.2. Antibodies and Reagents

Mouse monoclonal anti-HCV core antibody and rapamycin were purchased from Thermo Fisher Scientific (Rockford, IL, USA). Mouse monoclonal anti-SREBP-1c, mouse monoclonal anti-fatty acid synthase (FAS), and goat polyclonal anti-stearoyl CoA desaturase (SCD) were obtained from Santa Cruz Biotechnology (Santa Cruz, CA, USA). Mouse monoclonal anti-HCV NS5A antibody was obtained from Virogen (Watertown, MA, USA). Polyclonal antibodies specific to mTOR, lipin-1, and acetyl CoA carboxylase (ACACA) were purchased from Cell Signaling Technology, Inc. (Danvers, MA, USA). Scrambled miRNA, miR-99a-5p mimics, and miR-99a-5p inhibitor were synthesized and obtained from Genolution Pharmaceuticals (Seoul, Korea). We used miR-99a-5p because miR-99a-5p is a dominant arm among the two strands of miRNA duplex. Scrambled small interfering RNA (siRNA) and siRNA targeting mTOR were obtained from Cell Signaling Technology, Inc. Cells were transfected with miRNA mimics or siRNAs using lipofectamine^TM^ 2000 (Thermo-Fisher Scientific, MA, USA), according to the manufacturer’s instructions at a final concentration of 50 nM. pcDNA3_flag_mTOR and pcDNA3.1_flag_SREBP-1c were purchased from Addgene (Cambridge, MA, USA).

### 2.3. Cell-Culture-Derived HCV (HCVcc) and HCV Replicon System

Huh-7 cells were provided by Dr. Jane C. Moores (The Regent of the University of California, Oakland, CA, USA). The cells were maintained in Dulbecco’s modified Eagle’s medium (DMEM; Invitrogen, Carlsbad, CA, USA), supplemented with 10% fetal bovine serum, antibiotics (100 μg/mL of penicillin and 0.25 μg/mL of streptomycin), and 10 mM HEPES, in a humidified incubator at 37 °C in 5% CO_2_. Full-length, infectious HCV RNA of genotype 2a HCV clone JFH1 were prepared by in vitro transcription and electroporated into Huh-7 cells to obtain HCVcc, as previously described [9]. Huh-7 cells were infected with HCVcc at a multiplicity of infection (MOI) value of 1 by adsorption for 6 h with periodic rocking.

An HCV sub-genomic replicon (SGR) construct (pSGR-JFH1) and HCV full-genomic replicon (FGR) construct (pFGR-JFH1) were kindly provided by Dr. Takaji Wakita (National Institute of Infectious Disease, Tokyo, Japan). Huh-7 cell-derived cell lines, containing the HCV SGR or HCV FGR constructs, were established by transfection of in vitro transcribed HCV sub-genomic or HCV full-genomic RNA and selection using G418 sulfate (500 μg/mL).

### 2.4. RNA Extraction, Complementary DNA (cDNA) Synthesis, and Real-Time Quantitative Polymerase Chain Reaction (PCR)

Total RNA isolation, cDNA synthesis, and TaqMan real-time quantitative PCR analysis were performed, as previously described [9]. TaqMan gene expression assays (Applied Biosystems, Foster city, CA, USA) were used to determine the mRNA level of the target genes. The assay ID of each gene was as follows: mTOR (Hs00234508_m1), SREBP-1c (Hs01088679_m1), ACACA (Hs01046047_m1), FAS (Hs01005622_m1), SCD (Hs01682761_m1), and glyceraldehyde 3-phosphate dehydrogenase (GAPDH) (Hs02758991_m1). In addition, expression of miR-99a in HCVcc-infected Huh-7 cells and sera of patients with chronic HCV infection were measured using the TaqMan microRNA Assay kit (Applied Biosystems); each sample was normalized to the expression of U6 miRNA.

### 2.5. Immunoblotting

Cells were lysed with RIPA buffer that contained phosphatase inhibitors. Total protein content was determined using a Bradford protein assay kit (Bio-Rad Laboratories, Hercules, CA, USA). The density of each band was analyzed using the Multi Gauge V3.0 program as previously described (Fujifilm, Tokyo, Japan) [10].

### 2.6. Intracellular LDs Quantification

After fixation, the cells were stained with Nile Red (0.5 mg/mL) and 4,6-diamidino-2-phenylindole (DAPI, 1 mg/mL) (Sigma–Aldrich). After staining, intracellular LDs were quantified by measuring the density of fluorescence with a microplate reader (Molecular Devices, Sunnyvale, CA, USA), and the results were normalized to the cellular DAPI content. The distribution of lipids in cells was observed under an LSM 510 inverted laser-scanning confocal microscope (Carl Zeiss, Jena, Germany).

### 2.7. Statistical Analysis

All data are represented as mean ± standard error of at least three separate experiments. For comparison of multiple groups, one-way analysis of variance with Turkey’s post hoc test was used to define the statistical significance of differences among the groups. Unpaired t-tests or repeated-measures ANOVA were used to assess for statistical differences. An asterisk was used to express the statistical significance of differences among groups (* *p* <0.05, ** *p* <0.01, *** *p* <0.001).

## 3. Results

### 3.1. miR-99a Levels Were Down-Regulated in HCV-Infected Cells

**Figure 1:**
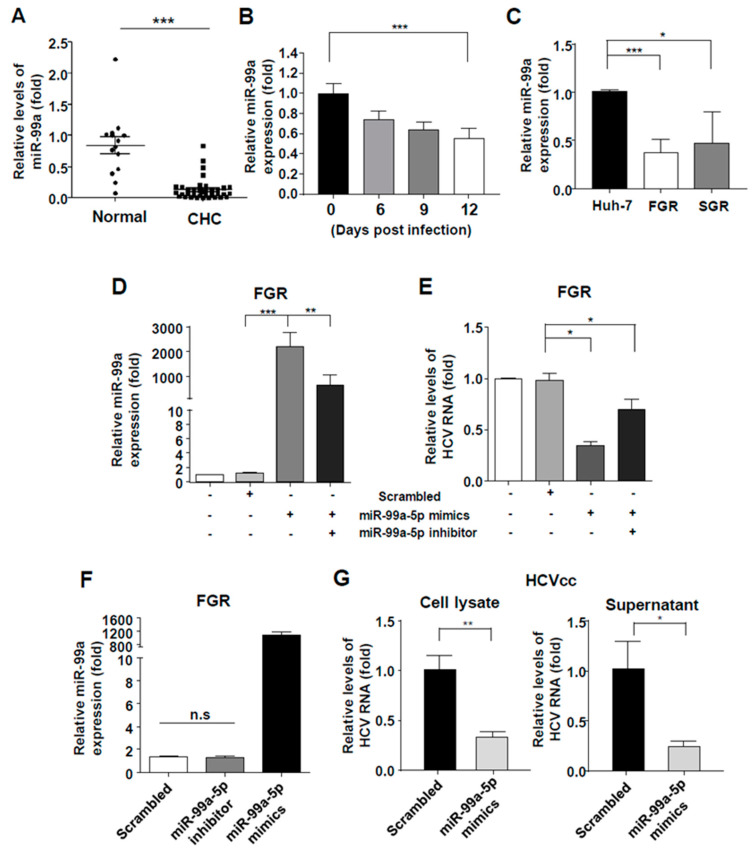
Overexpression of miR-99a-5p attenuates HCV replication. (**A**) Expression levels of miR-99a in the sera of 37 patients with chronic hepatitis C virus (HCV) infection and 14 healthy donors. Bar graphs represent the means ± s.d. Unpaired t-tests were performed. *** *p* < 0.001; (**B**) Serial levels of miR-99a after cell culture-derived HCV (HCVcc) infection in Huh-7 cells (MOI = 1). Means ± s.e.m. are shown (*n* = 5). Repeated-measures ANOVA was performed. *** *p* < 0.001; (**C**) baseline miR-99a expression in parental Huh-7 cells, full-genomic replicon (FGR) cells, and sub-genomic replicon (SGR) cells. Means ± s.e.m. are shown (*n* = 3). Unpaired t-tests were performed. * *p* < 0.05, *** *p* < 0.001; (**D**–**F**) miR-99a levels (**D**,**F**) and HCV RNA levels (**E**) in miR-99a-5p mimics- or miR-99a-5p inhibitor-transfected FGR cells after 72 h. Means ± s.e.m. are shown (*n* = 5). Unpaired t-tests were performed. * *p* < 0.05, ** *p* < 0.01, *** *p* < 0.001; (**G**) HCV RNA levels in cell lysate and culture supernatant in HCVcc-infected Huh-7 cells (MOI = 1, 5 days after infection), 48 h after transfection of mock or miR-99a-5p mimics. Means ± s.e.m. are shown (*n* = 3). Unpaired t-tests were performed. * *p* < 0.05, ** *p* < 0.01.

Expression levels of miR-99a in the sera of 37 patients with chronic HCV infection and 14 healthy donors were analyzed. The relative expression of miR-99a was significantly lower in the sera from the chronic HCV infection patients than in those from subjects without viral hepatitis (Figure 1A). Moreover, as shown in Figure 1B, expression of miR-99a in Huh-7 cells infected with HCVcc steadily decreased from 25% on day 6 to over 40% on day 12. To confirm the down-regulation of miR-99a in HCV-replicating cells, we assessed miR-99a expression in genotype 2a HCV FGR and SGR cells. The endogenous expression levels of miR-99a were significantly lower in these cells than in the parental Huh-7 cells (Figure 1C).

### 3.2. Overexpression of miR-99a-5p Attenuated HCV Replication

Next, we observed the effects of the overexpression of miR-99a-5p in HCV-replicating cells. The expression of miR-99a increased more robustly in miR-99a-5p mimic-transfected FGR cells than in scrambled miRNA-transfected cells. Simultaneous transfection of miR-99a-5p and miR-99a inhibitors significantly reduced the expression of transfected miR-99a (Figure 1D). To examine the effect of miR-99a on HCV replication, FGR cells were transfected with miR-99a-5p mimics. As shown in Figure 1F, miR-99a-5p transfection resulted in an approximately 80% decrease in the levels of intracellular HCV RNA in FGR cells (Figure 1E). HCV RNA levels restored when miR-99a-5p mimics and inhibitors were simultaneously transfected in FGR cells (Figure 1E). However, the miR-99a-5p inhibitor did not significantly reduce the miR-99a level in FGR cells because of the low level of endogenous miR-99a in these HCV-replicating cells (Figure 1F). In HCVcc-infected Huh-7 cells, transfection of miR-99a mimics significantly reduced both the levels of intracellular and secreted HCV RNA levels (Figure 1G). 

### 3.3. mTOR and Its Downstream Signal Was Targeted by miR-99a in HCV-Replicating Cells

**Figure 2:**
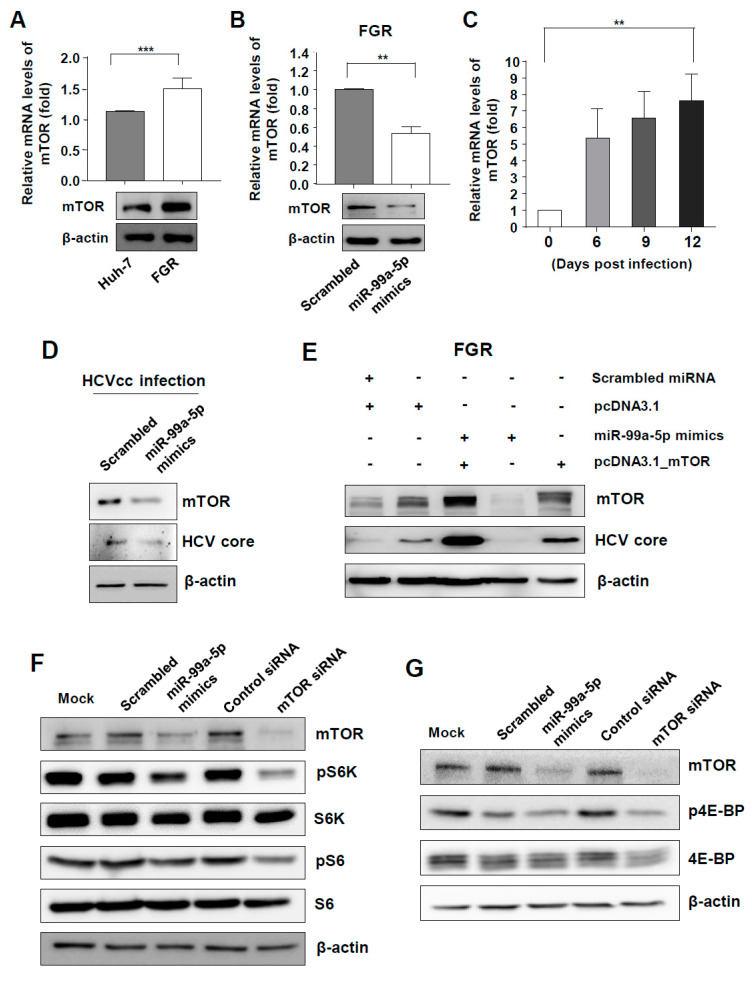
miR-99a causes inefficient HCV replication by regulating mammalian target of rapamycin (mTOR)/ sterol regulatory element binding protein (SREBP)-1c. (**A**) Expression levels of mTOR in parental Huh-7 and FGR cells. Means ± s.e.m. are shown (*n* = 3). Unpaired t-tests were performed. *** *p* < 0.001; (**B**) expression levels of mTOR in FGR cells 48 h after miR-99a-5p transfection. Means ± s.e.m. are shown (*n* = 3). Unpaired t-tests were performed. ** *p* < 0.01; (**C**) serial levels of mTOR expression after HCVcc infection in Huh-7 cells (MOI = 1). Repeated-measures ANOVA was performed. ** *p* < 0.01; (**D**) HCV core and mTOR protein levels 48 h after miR-99a-5p mimics transfection. Data from three independent experiments are presented; (**E**) HCV core and mTOR protein levels 48 h after miR-99a-5p mimics and/or pcDNA3.1_mTOR transfection. Data from three independent experiments are presented; (**F**) downstream signals (S6K) of mTORC1 after miR-99a-5p mimics or si-mTOR transfection in FGR cells. Data from three independent experiments are presented; (**G**) downstream signals (4E-BP) of mTORC1 after miR-99a-5p mimics or si-mTOR transfection in FGR cells. Data from three independent experiments are presented.

Using the in silico analysis tools miRanda and TargetScan, we confirmed that miR-99a targets the 3’ UTR of mTOR with a high binding score. Both mRNA and protein levels of mTOR were more up-regulated in FGR cells than in parental Huh-7 cells (Figure 2A). After transfection of miR-99a-5p mimics in these cells, both mRNA and protein levels of mTOR significantly decreased (Figure 2B). mTOR expression also increased in Huh-7 cells after HCVcc infection (Figure 2C), and transfection of miR-99a-5p mimics in HCVcc-infected Huh-7 cells caused down-regulation of both mTOR and HCV core protein (Figure 2D). Importantly, overexpression of mTOR gene rescued HCV replication in miR-99a-5p-transfected FGR cells, suggesting that mTOR is responsible for miR-99a-mediated restriction of HCV replication (Figure 2E).

We then assessed the effect of miR-99a on downstream signaling of mTORC1 in HCV-replicating cells. miR-99a-5p mimics reduced the level of phosphorylated S6K in FGR cells compared with cells transfected with scrambled miRNA. As a positive control, si-mTOR also down-regulated the level of phosphorylated S6K (Figure 2F). When protein expression of mTOR decreased by miR-99a-5p transfection in FGR cells, levels of phosphorylated 4E-BP were also decreased (Figure 2G). Collectively, these data suggest that mTOR is a target of miR-99a, and its downstream signaling is also affected by miR-99a.

### 3.4. miR-99a-5p Transfection Attenuated Intracellular Lipid Accumulation via the mTORC1-SREBP Axis

**Figure 3:**
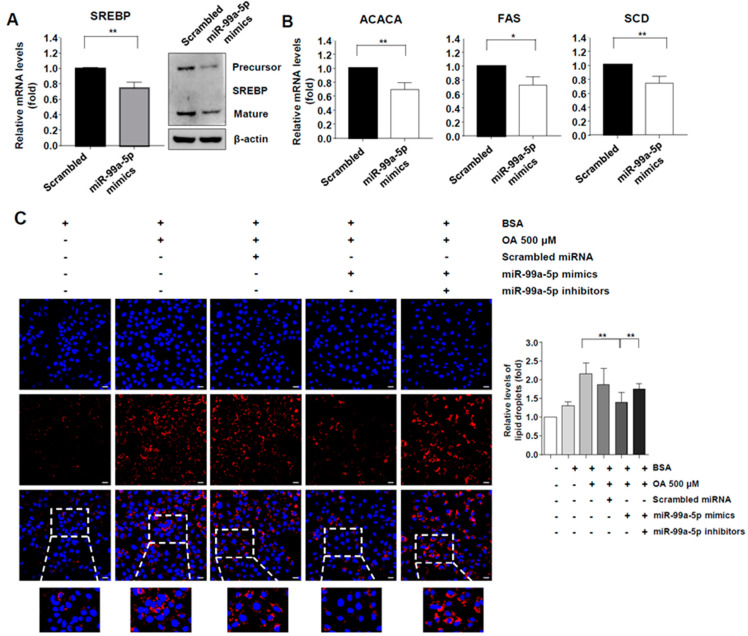
miR-99a ameliorates intracellular lipid accumulation by regulating mTOR/SREBP-1c. (**A**) Expression levels of SREBP1-c in FGR cells 48 h after miR-99a-5p transfection. Means ± s.e.m. are shown (*n* = 3). Unpaired t-tests were performed. ** *p* < 0.01; (**B**) expression levels of acetyl CoA carboxylase (ACACA), fatty acid synthase (FAS), and stearoyl CoA desaturase (SCD) in FGR cells 48 h after miR-99a-5p transfection. Means ± s.e.m. are shown (*n* = 3). Unpaired t-tests were performed. * *p* < 0.05. ** *p* < 0.01; (**C**) amount of intracellular lipid accumulation after OA (500 uM) treatment and miR-99a-5p mimics or inhibitors transfection. Data from two independent experiments are presented. Unpaired t-tests were performed. ** *p* < 0.01.

As noted previously, SREBP-1c is known to induce de novo lipogenesis to generate free fatty acids [11]. HCV infection can increase intrahepatic lipogenesis via SREBP activation [12]. Moreover, mTORC1 and S6K1 have been reported to promote processing and activation of SREBP-1c [13]. Therefore, we investigated the effects of miR-99a-5p transfection on de novo lipogenesis in HCV-replicating cells. We confirmed that the expression levels of both mRNA and protein of SREBP-1c were significantly down-regulated in FGR cells that were transfected with miR-99a-5p mimics (Figure 3A). The mRNA levels of SREBP-1c target genes, ACACA, FAS, and SCD were down-regulated after miR-99a-5p overexpression (Figure 3B). Taken together, these results demonstrated that miR-99a regulated the expression of SREBP-1c and its target genes.

We further investigated whether miR-99a overexpression affected intracellular lipid accumulation. FGR cells were transfected with miR-99a-5p mimics or co-transfected with miR-99a-5p mimics and miR-99a-5p inhibitors. Treatment with oleic acids (OA) increased intracellular lipid levels in Huh-7 cells up to approximately 200% (Figure 3C). However, the level of accumulated intracellular lipid after treatment with OA decreased significantly in cells that overexpressed miR-99a-5p mimics compared with cells treated with scrambled miRNAs (Figure 3C). Moreover, the level of accumulated lipids increased in cells co-transfected with miR-99a-5p mimics and inhibitors compared with cells transfected only with miR-99a-5p mimics (Figure 3C). Collectively, these results indicated that miR-99a ameliorated intracellular lipid accumulation by regulating the expression of SREBP-1c and its target genes

## 4. Discussion

In this report, we clearly demonstrated that miR-99a ameliorate intracellular lipid accumulation by regulating the expression of mTOR, causing down-regulation of SREBP-1c and its target genes. This attenuated lipogenesis results in the inefficient replication and packaging of HCV. Our data show the critical association between HCV infection and intracellular LDs, and miRNAs that can regulate the accumulation of intracellular triglycerides might be considered as host targets with both anti-lipogenic and anti-HCV activities.

Previous reports demonstrated that various cellular miRNAs can interact with viral genome and inhibit HCV replication [14,15]. Furthermore, deregulation of many miRNAs can be associated with different stages of HCV-induced HCC [16]. More recent studies have identified miRNAs that regulate both intrahepatic lipid metabolism and HCV infection [17]. miR-27a was reported to target retinoid X receptor alpha and regulate fat metabolism, and it also plays an important role in HCV replication [18]. miR-122, which is a well-known liver-specific miRNA, also takes a critical role in regulating hepatic lipid metabolism. Liver-specific deletion of miR-122 resulted in a dramatic decrease in serum triglyceride and cholesterol levels [17]. miR-122 is also essential for HCV replication in hepatocytes, and targeting miR-122 led to the suppression of the viral replication in HCV-infected chimpanzees and patients [19,20]. Until now, miR-99a has been reported only as a suppressor of tumor invasion and metastasis in various types of cancers [21,22,23]. Our study is the first to report that miR-99a also has anti-lipogenic and anti-HCV function in hepatocytes, similar to that of miR-122 and miR-27a. Although we did not perform a prospective study, we assume that HCV infection led to the dramatic decrease in the levels of serum miR-99a in the infected patients. Previous reports have demonstrated that miR-99a was down-regulated in HCC tissues compared with non-tumor tissues [24]. Therefore, HCV-induced down-regulation of miR-99a may be more pronounced in the human liver than that in the in vitro cell culture system.

Previous reports demonstrated the association between miR-99a and mTOR [21,22,23]. miR-99a functions as a tumor suppressor in various cancers by targeting the mTOR signaling pathway [21,22,23]. Regarding HCV infection, miR-99a activates mTOR signaling to block apoptosis of the infected cells [6]. Recent reports have demonstrated that mTOR inhibitors inhibit HCV replication without affecting the cell viability [6]. As mentioned in the previous sections, mTOR is critically associated with hepatic lipid metabolism. The stability, abundance, and processing of SREBP-1c are increased on stimulation of the mTORC1 signaling pathway, leading to an increase in the transcription of lipogenic genes [11,13]. Therefore, targeting the mTOR pathway can result in both anti-lipogenic and anti-HCV effects. Although anti-HCV activity by miR-99a may be an independent process from the decreased de novo lipogenesis, increasing the abundance of miR-99a can be a new therapeutic option for these mTOR-mediated pathologic conditions.

In conclusion, our data reveal that miR-99a ameliorate intracellular lipid accumulation by regulating the expression of mTOR, causing down-regulation of SREBP-1c, and inefficient replication and packaging of HCV. Future studies will confirm the utility of this miRNA as a biomarker for HCV-induced hepatic steatosis or non-alcoholic fatty liver disease progression.

## Abbreviations

HCV	hepatitis C virus
HCC	hepatocellular carcinoma
LD	lipid droplets
mTOR	mammalian target of rapamycin
S6K	ribosomal protein S6 kinases
eIF4E	eukaryotic initiation factor 4E
4E-BP	eIF4E-binding protein
SREBP	sterol regulatory element binding proteins
miR-99a	miRNA-99a
FAS	fatty acid synthase
SCD	stearoyl CoA desaturase
ACACA	acetyl CoA carboxylase
siRNA	small interfering RNA
HCVcc	cell culture-derived HCV
MOI	multiplicity of infection
SGR	sub-genomic replicon
FGR	full-genomic replicon
cDNA	complementary DNA
PCR	polymerase chain reaction
GAPDH	glyceraldehyde 3-phosphate dehydrogenase
DAPI	4,6-diamidino-2-phenylindole
OA	oleic acids.

## References

[1] Sung P.S., Shin E.C. (2020). Interferon response in hepatitis C virus-infected hepatocytes: Issues to consider in the era of direct-acting antivirals. Int. J. Mol. Sci..

[2] Marascio N., Quirino A., Barreca G.S., Galati L., Costa C., Pisani V., Mazzitelli M., Matera G., Liberto M.C., Foca A. (2019). Discussion on critical points for a tailored therapy to cure hepatitis C virus infection. Clin. Mol. Hepatol..

[3] Park S.H., Plank L.D., Suk K.T., Park Y.E., Lee J., Choi J.H., Heo N.Y., Park J., Kim T.O., Moon Y.S. (2020). Trends in the prevalence of chronic liver disease in the Korean adult population, 1998–2017. Clin. Mol. Hepatol..

[4] Vieyres G., Pietschmann T. (2019). HCV pit stop at the lipid droplet: Refuel lipids and put on a lipoprotein coat before exit. Cells.

[5] Meyers N.L., Fontaine K.A., Kumar G.R., Ott M. (2016). Entangled in a membranous web: ER and lipid droplet reorganization during hepatitis C virus infection. Curr. Opin. Cell Biol..

[6] Stöhr S., Costa R., Sandmann L., Westhaus S., Pfaender S., Dazert E., Meuleman P., Vondran F.W., Manns M.P., Steinmann E. (2016). Host cell mTORC1 is required for HCV RNA replication. Gut.

[7] Laplante M., Sabatini D.M. (2012). mTOR signaling in growth control and disease. Cell.

[8] Porstmann T., Santos C.R., Griffiths B., Cully M., Wu M., Leevers S., Griffiths J.R., Chung Y.L., Schulze A. (2008). SREBP activity is regulated by mTORC1 and contributes to Akt-dependent cell growth. Cell Metab..

[9] Sung P.S., Lee E.B., Park D.J., Lozada A., Jang J.W., Bae S.H., Choi J.Y., Yoon S.K. (2018). Interferon-free treatment for hepatitis C virus infection induces normalization of extrahepatic type I interferon signaling. Clin. Mol. Hepatol..

[10] Park D.J., Sung P.S., Kim J.H., Lee G.W., Jang J.W., Jung E.S., Bae S.H., Choi J.Y., Yoon S.K. (2020). EpCAM-high liver cancer stem cells resist natural killer cell-mediated cytotoxicity by upregulating CEACAM1. J. Immunother Cancer.

[11] Owen J.L., Zhang Y., Bae S.H., Farooqi M.S., Liang G., Hammer R.E., Goldstein J.L., Brown M.S. (2012). Insulin stimulation of SREBP-1c processing in transgenic rat hepatocytes requires p70 S6-kinase. Proc. Natl. Acad. Sci. USA.

[12] Li Q., Pene V., Krishnamurthy S., Cha H., Liang T.J. (2013). Hepatitis C virus infection activates an innate pathway involving IKK-alpha in lipogenesis and viral assembly. Nat. Med..

[13] Laplante M., Sabatini D.M. (2013). Regulation of mTORC1 and its impact on gene expression at a glance. J. Cell Sci..

[14] Piedade D., Azevedo-Pereira J.M. (2016). MicroRNAs, HIV and HCV: A complex relation towards pathology. Rev. Med. Virol..

[15] Sedano C.D., Sarnow P. (2015). Interaction of host cell microRNAs with the HCV RNA genome during infection of liver cells. Semin. Liver Dis..

[16] Sadri Nahand J., Bokharaei-Salim F., Salmaninejad A., Nesaei A., Mohajeri F., Moshtzan A., Tabibzadeh A., Karimzadeh M., Moghoofei M., Marjani A. (2019). microRNAs: Key players in virus-associated hepatocellular carcinoma. J. Cell Physiol..

[17] Yang Z., Cappello T., Wang L. (2015). Emerging role of microRNAs in lipid metabolism. Acta Pharm. Sin. B.

[18] Shirasaki T., Honda M., Shimakami T., Horii R., Yamashita T., Sakai Y., Sakai A., Okada H., Watanabe R., Murakami S. (2013). MicroRNA-27a regulates lipid metabolism and inhibits hepatitis C virus replication in human hepatoma cells. J. Virol..

[19] Lanford R.E., Hildebrandt-Eriksen E.S., Petri A., Persson R., Lindow M., Munk M.E., Kauppinen S., Orum H. (2010). Therapeutic silencing of microRNA-122 in primates with chronic hepatitis C virus infection. Science.

[20] Janssen H.L., Reesink H.W., Lawitz E.J., Zeuzem S., Rodriguez-Torres M., Patel K., van der Meer A.J., Patick A.K., Chen A., Zhou Y. (2013). Treatment of HCV infection by targeting microRNA. N. Engl. J. Med..

[21] Yin H., Ma J., Chen L., Piao S., Zhang Y., Zhang S., Ma H., Li Y., Qu Y., Wang X. (2018). MiR-99a enhances the radiation sensitivity of non-small cell lung cancer by targeting mTOR. Cell. Physiol. Biochem..

[22] Zhu Y., Zhang S., Li Z., Wang H., Li Z., Hu Y., Chen H., Zhang X., Cui L., Zhang J. (2019). miR-125b-5p and miR-99a-5p downregulate human gammadelta T-cell activation and cytotoxicity. Cell. Mol. Immunol..

[23] Wu S.H., Han L., Lu B.C., Wang H.Y., Zheng C.P. (2019). MiR-99a inhibits cell proliferation of nasopharyngeal carcinoma by targeting mTOR and serves as a prognostic factor. Eur. Rev. Med. Pharmacol. Sci..

[24] Hou J., Lin L., Zhou W., Wang Z., Ding G., Dong Q., Qin L., Wu X., Zheng Y., Yang Y. (2011). Identification of miRNomes in human liver and hepatocellular carcinoma reveals miR-199a/b-3p as therapeutic target for hepatocellular carcinoma. Cancer Cell.

